# Vaccine Breakthrough Infections Among Healthcare Workers in a COVID-19-Designated Tertiary Care Government Hospital in Sikkim

**DOI:** 10.7759/cureus.46752

**Published:** 2023-10-09

**Authors:** Shrijana Gurung, Ekta Tewari, Pooja Pradhan, Tsultem D Bhutia, Tashi P Chhophel, Maricca M Rasaily, Mani Gurung, Ashish Rai, Manoj Sarda, Birendra Gurung, Priya D Pradhan, Dhruva K Sharma

**Affiliations:** 1 Virology, Sir Thutob Namgyal Memorial Hospital, Government of Sikkim, Gangtok, IND; 2 Paediatrics, Sir Thutob Namgyal Memorial Hospital, Government of Sikkim, Gangtok, IND; 3 Medicine, Apollo Indraprastha Hospital, New Delhi, IND; 4 Obstetrics and Gynaecology, Sir Thutob Namgyal Memorial Hospital, Government of Sikkim, Gangtok, IND; 5 Radiation Oncology, Sir Thutob Namgyal Memorial Hospital, Government of Sikkim, Gangtok, IND; 6 Surgery, Sir Thutob Namgyal Memorial Hospital, Government of Sikkim, Gangtok, IND; 7 Pathology, Sir Thutob Namgyal Memorial Hospital, Government of Sikkim, Gangtok, IND; 8 Pharmacology and Therapeutics, Sikkim Manipal Institute of Medical Sciences, Sikkim Manipal University, Gangtok, IND

**Keywords:** sikkim, health care workers, breakthrough infection, sars-cov-2, covid-19

## Abstract

Introduction

Since the emergence of the coronavirus disease 2019 (COVID-19) virus at the beginning of 2020, the world has gone through various waves of pandemics. The health care workers (HCWs) or the COVID warriors as they were termed were the first line of defense against the virus. They were armed with personal protective equipment and prophylactic doses of the COVID-19 vaccine. Despite these precautions, some of the HCWs still contracted the disease and a few others succumbed to it. The objective of this study was to estimate the prevalence of COVID-19 infections and vaccine breakthrough infections (BTIs) in HCWs after receiving the COVID-19 vaccine during the second wave of the pandemic.

Methods

This was a cross-sectional, hospital-based study conducted over a period of four months from September 2021 to December 2021 on HCWs aged 18 years and above working at the COVID-19-designated tertiary care government hospital in Sikkim. A structured coded questionnaire with no patient identifiers was used to gather details on demographics, vaccination history, breakthrough infection, and other social details. HCWs who had received at least one dose of the COVID-19 vaccine at the time of initiation of the study and were >18 years of age were included in this study.

Results

A total of 678 HCWs were screened, out of which 229 (33%) participants tested positive for COVID-19 and the rest of the participants (455; 67%) tested negative. COVID-19 infections and vaccine BTIs (COVID-19 infection >14 days after the second vaccination) were recorded and 137 (20%) respondents had a post-vaccination COVID-19 infection out of which 115 (18.5%) were BTI. The majority of the participants were females and of the age group of 26-35 years. The correlation of COVID-19 infections with the dose gap between vaccination, gender, age, profession, department, area posted during COVID duty, cycles of duty performed, hospitalization due to infection, influenza vaccination, and comorbidity was analyzed.

Conclusion

COVID-19 vaccines are disease-modifying and they decrease the severity of BTIs in HCWs. Pandemics and outbreaks cannot be predicted; therefore, it becomes very important to have healthy frontline workers who are constantly exposed to infectious agents. Monitoring of health and surveillance of infectious diseases among the HCWs should be encouraged.

## Introduction

Severe acute respiratory syndrome virus 2 (SARS CoV-2) was responsible for the coronavirus disease 2019 (COVID-19). It is an RNA virus of the family Coronaviridae and order Nidovirales, which infects both humans and animals [1]. To combat the global spread and mortality due to SARS-CoV-2, 165 candidate vaccines were approved by the WHO on July 31, 2020 [2]. Out of these candidate vaccines, the Astra Zeneca/Oxford viral vector vaccine ChAdOx1-S (known as “Covishield” in India) was tested and manufactured by the Serum Institute of India (Pune, India) [3]. Two types of indigenous vaccines, Covishield and Covaxin, were used to start the COVID-19 vaccination program in India on January 6, 2021 [4]. The initial recipients of vaccination included healthcare workers (HCWs), front-line workers, senior citizens, and those with associated co-morbidities [4].

In Sikkim, a vaccination drive with Covishield was initiated along with the rest of the country for all HCWs. The state had provided 5,54,656 with the second dose and 2,13,944 precautionary doses of the COVID vaccine as of September 25, 2023 [5]. Covishield is a recombinant inactivated virus vaccine that can mount an immune response against the COVID-19 infection [6,7]. A multicentric study conducted by the Indian Council of Medical Research (ICMR) declared that Covishield was 85% effective against COVID-19 infection [8]. They reduce the risk of severe COVID-19 infections but vaccine breakthrough infections (BTIs) have been known to occur [4,8]. BTI has been defined as the identification of SARS-CoV-2 or its antigen in respiratory specimens obtained from a person 14 days after receiving all doses of the COVID-19 vaccine [9]. A myriad of factors have been implicated in causing BTI. The properties of the virus itself like the variant of the virus, initial viral load at the time of infection, length of incubation period [10], host factors like immunocompromised condition, and properties of the vaccine like the type of vaccine given, dose interval, route of administration, all have a role to play in the outcome of BTI [10].

Studies done in India among HCWs have found BTI to vary from 5.08% to 76.4% [11,12]. The primary objective of the study was to estimate the prevalence of COVID-19 infection and BTI among the HCWs who had received the Covishield vaccine.

## Materials and methods

Ethics and approval

Ethical clearance was obtained from the institutional ethics committee of Sir Thutob Namgyal Memorial Hospital, vide order no 04/IEC/STNMH/21. Permission was also obtained from the Health and Family Welfare Department, Government of Sikkim, to carry out the study and publish the results. Informed written consent was taken before collecting patients information and only those volunteers that gave their consent were included in the study.

Study setting and sample size

This was a cross-sectional, hospital-based study that was conducted for four months between September 2021 and December 2021. The study participants were HCWs aged 18 years and above working at the tertiary care government referral hospital, which was also the only dedicated COVID-19 hospital in the state during the pandemic. It is a 1,000-bed hospital that receives referrals from all six districts. A structured coded questionnaire was used without participant identifiers to determine the prevalence of COVID-19 infection and breakthrough infection and to correlate social, demographic, economic, health, and environmental factors with COVID-19 infection.

Assuming that 17% of the HCWs in the hospital have COVID-19, a design effect (DEFF) of 2, and an expected response rate of 70%, the study would require a sample size of 620 to estimate the expected proportion with 5% absolute precision and 95% confidence [13].

Based on the above sample size calculation, a total of 700 questionnaires were distributed amongst the HCWs out of which 684 HCWs consented to participate in this study. Six respondents were not vaccinated and hence only 678 responses were chosen for further analysis.

Inclusion criteria

HCWs who had received at least one dose of the COVID-19 vaccine at the time of initiation of the study, who were >18 years of age and working in the hospital were included in this study.

Exclusion criteria

HCWs who had not received any COVID-19 vaccine and those who did not give their consent were excluded.

Statistical analysis

Statistical Package for the Social Sciences (SPSS) version 27 (IBM Corp., Armonk, NY) and Microsoft Excel version 2007 (Microsoft Corporation, Redmond, WA) were used for statistical analysis of the data. The association of various variables with the prevalence of COVID-19 infections was analyzed using the chi-squared test (χ 2), direct logistic regression, and Pearson’s correlation test (r), with a significance value of p ≤ 0.05.

## Results

Infection and breakthrough infection

A total of 678 HCWs were screened, out of which 226 (33%) participants tested positive for COVID-19 and the rest of the participants (452; 67%) tested negative (Table 1).

**Table 1:** Characteristics of the participants and their association with COVID-19 and breakthrough infections p-value <0.05: significant; n: total number of cases

Variables	COVID-19 Infections			Breakthrough Infections
	Yes, 226 (33%)		No, 452 (67%)	Yes, 115 (18.5%)
Gender, n (%), p-value 0.216				
Females, 502	174 (34.7%)		328 (65.3%)	84 (73%)
Males, 176	52 (29.5%)		124 (70.5%)	31 (27%)
Age group, n (%), p-value 0.087				
18-25, 81	20 (24.7%)		61 (75.3%)	10 (8.7%)
26-35, 295	99 (33.6%)		196 (66.4%)	50 (43.5%)
36-45, 147	48 (32.7%)		99 (67.3%)	27 (23.5%)
46-55, 134	51 (38.1%)		83 (61.9%)	25 (21.7%)
56-65, 21	8 (38.1%)		13 (61.9%)	3 (2.6%)
Profession, n (%), p-value <0.001				
Doctors, 127	50 (39.4%)		77 (60.6%)	27 (23.5%)
Nurses, 289	114 (39.4%)		175 (60.6%)	57 (49.6%)
Clinico-supportive staff, 142	27 (19%)		115 (81%)	14 (12.2%)
Administrative staff, 45	16 (35.6%)		29 (64.4%)	9 (7.8%)
Ward/lab attendants, 51	13 (25.5%)		38 (74.5%)	6(5.2%)
Sanitary attendants, 9	4 (44.4%)		5 (55.6%)	2 (1.7%)
Supportive staff, 15	2 (13.3%)		13 (86.7%)	0
Vaccination, n (%), p-value 0.497				
Single dose, 58	17 (29%)		41 (71%)	
Double dose, 620	209 (34%)		411 (66%)	
Cycles of COVID duty, n (%), p-value 0.001				
None, 251	61 (24.3%)		190 (75.7%)	28 (24.3%)
1-5 cycles, 310	121 (39%)		189 (61%)	63 (54.8%)
>5 cycles, 117	44 (37.6%)		73 (62.4%)	24 (20.9%)
Area posted, n (%), p-value 0.275				
Administrative areas, 7	1 (14.3%)		6 (85.7%)	1 (0.9%)
Diagnostic area, 30	6 (20%)		24 (80%)	3 (2.6%)
Direct patient care, 282	116 (41.1%)		166 (58.9%)	64 (55.7%)
General wards, 56	20 (35.7%)		36 (64.3%)	7 (6%)
Non-clinical areas, 3	1 (33.3%)		2 (66.7%)	1 (0.9%)
OT/ICU, 55	21 (38.2%)		34 (61.8%)	11 (9.6%)
None, 245	61 (24.9%)		184 (75.1%)	28 (24.3%)
Co-morbidity, n (%), p-value 0.114				
None, 596		187 (31.4%)	409 (68.6%)	91 (79.1)
Cancer, 1		1 (100%)	0	0
Hypertension, 43		22 (51.2%)	21 (48.8%)	13 (11.3%)
Diabetes mellitus, 28		14 (50%)	14 (50%)	10 (8.7%)
Chronic kidney disease 1		0	1 (100%)	0
On immunosuppressant, 5		1 (20%)	4 (80%)	0
Others, 4		1 (25%)	3 (75%)	1 (0.9%)
Departments, n (%), p-value 0.751				
Gastro, 15		4 (26.7%)	11 (73.3%)	1 (0.9%)
Administrative section, 36		13 (36.1%)	23 (63.9%)	8 (7%)
Anesthesia, 6		2 (33.3%)	4 (66.7%)	1 (0.9%)
Blood bank, 7		1 (14.3%)	6 (85.7%)	1 (0.9%)
Cardiology, 37		8 (21.6%)	29 (78.4%)	7 (6.1%)
Dental, 8		3 (37.5%)	5(62.5%)	0
Dermatology, 6		2 (33.3%)	4 (66.7%)	1 (0.9%)
ENT, 8		5 (62.5%)	3 (37.5%)	2 (17.4%)
Emergency, 26		8 (30.8%)	18 (69.2%)	3 (2.6%)
Eye, 9		2 (22.2%)	7 (77.8%)	0
General medicine, 59		20 (33.9%)	39 (66.1%)	8 (7%)
Gynecology, 38		24 (63.2%)	14 (36.8%)	19 (16.5%)
Laboratory, 119		28 (23.5%)	91 (76.5%)	13 (11.3%)
Oncology, 38		6 (15.8%)	32 (84.2%)	1 (0.9%)
Oncosurgery, 4		4 (100%)	0	3 (2.6%)
Orthopedics 33		10(30.3%)	23(69.7%)	0
Pediatrics 68		33(48.5%)	35(51.5%)	18 (15.7%)
Pediatric surgery, 5		2 (40%)	3 (60%)	0
Physiotherapy, 10		5 (50%)	5 (50%)	4 (3.5%)
Psychiatry, 28		11 (39.3%)	17 (60.7%)	5(4.3%)
Radiology, 35		7 (20%)	28 (80%)	3 (2.6%)
Surgery, 53		19 (35.8%)	34 (64.2%)	12 (10.4%)
Urban public health, 30		9 (30%)	21 (70%)	5 (4.3%)

The respondents were diagnosed positive for SARS-CoV-2 based on reports for reverse transcription-polymerase chain reaction (RT-PCR) (87%), rapid antigen test (RAT) (10%), CT scan (2%), and automated cartridge-based nucleic acid amplification test (NAAT) (1%). A total of 620 (91%) had received both doses of the Covishield vaccine (Table 1).

**Figure 1:**
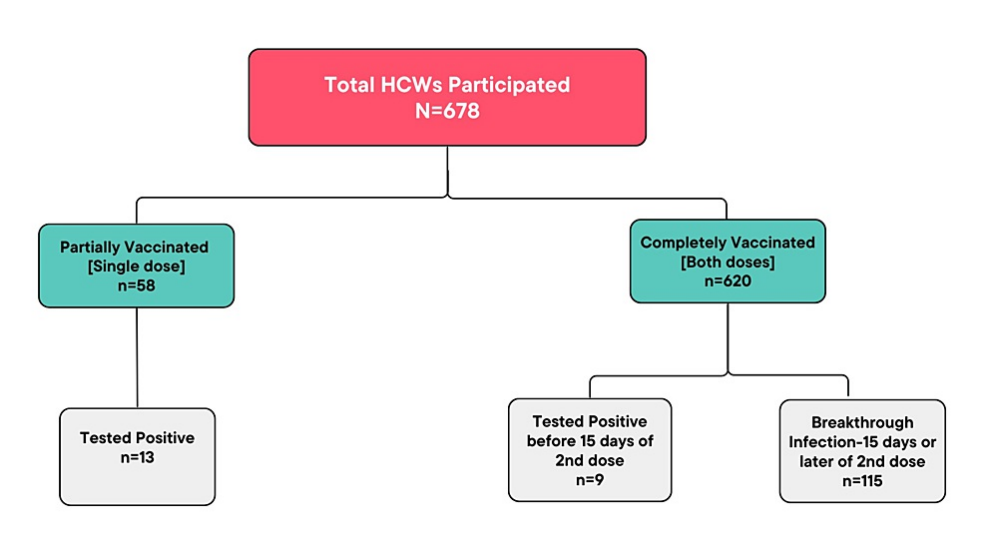
Flow chart depicting the study population and their COVID-19 status

Fifty-eight (58) respondents had received only one dose of the vaccine among which 13 tested positive for COVID-19, 620 had received both doses, and 124 were positive for COVID-19 infection (Table 2). Figure 1 shows a flow chart depicting vaccinated respondents.

**Table 2:** Summary of COVID-19 infections, vaccination status, and symptoms associated with infection BTI - breakthrough infection

Vaccinated	Vaccination doses		COVID-19 infections		Symptomatic cases		Asymptomatic cases		Pre-vaccination infections	Post-vaccination infections	BTI
	1st dose	2nd dose	1st dose	2nd dose	Post-Vaccine Infection	BTI	Post-Vaccine Infection	BTI			
678	58	620	13	124	122	101	15	14	89	137	115

Among the 226 COVID-19-positive vaccinated respondents, 89 (39%) tested positive before vaccination and the prevalence of COVID-19 infections among HCWs was lower after the first dose of vaccination 13 (6%) as compared to after the second dose (124; 55%) (Table 2).

The timeline of post-vaccination COVID-19 infections and BTIs (>14 days after the second vaccination) was recorded and 137/678 (20%) were post-vaccination COVID-19 cases out of which 115 (18.5%) were BTI (Table 2). Table 2 depicts the months when the HCWs with BTI turned positive for COVID-19 after two doses of vaccine. Forty-two (42, 37%) positive cases were recorded in the month of May 2021 (Table 3).

**Table 3:** Timeline of breakthrough infections

Months	No. of Breakthrough Infections
March 2021	1
April 2021	9
May 2021	43
June 2021	26
July 2021	17
August 2021	13
September 2021	5
November 2021	1
Total Breakthrough Infections	115

Spectrum of symptoms

The spectrum of symptoms related to COVID-19 infections recorded in vaccinated HCWs included cold and cough, headache, loose stools, and loss of smell and taste. One-hundred twenty-two (122) of 678 (18%) of the vaccinated HCWs were symptomatic and 15 of 678 (2%) were asymptomatic. Among the HCWs with BTI, 101/620 (16%) symptomatic and 14/620 (2%) asymptomatic cases were seen (Table 2).

Gender

Five-hundred two (502; 74%) of the participants in this study were females and 176 (26%) were males. A total of 174 (35%) females were COVID-19 positive; 68/174 (39%) before vaccination, 12/174 (7%) after the first dose, 92/174 (53%) after the second dose of vaccination, and 84/174 (48%) BTI (Table 1).

Fifty-two (52; 30%) males were found to be infected with COVID-19, 19 (37%) before vaccination, 32 (62%) after the second dose, and 31 (60%) were reported as BTI (Table 1).

Chi-square analysis revealed no significance between gender and the frequency of COVID-19 infections, χ2 (1, n = 678) = 1.3, p = 0.215.

Age 

The maximum number of respondents in this study belonged to the age group of 26-35 years. Overall post-vaccination COVID-19 infections and BTIs were seen to be highest in the age group of 26-35 (Table 1). No statistical significance was seen in the prevalence of post-vaccination infection among the different age groups (p=0.087) (Table 1).

Profession and departments

**Figure 2:**
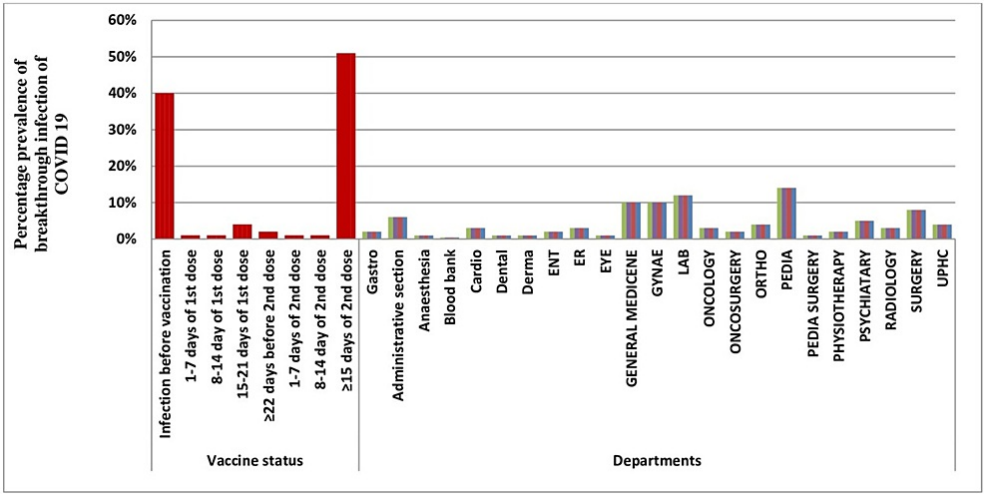
Graph showing COVID-19 infection in association with vaccine days and with the departments

Different categories of HCWs were doctors, nurses, clinic supportive staff, administrative staff, ward/lab attendants, sanitary attendants, and supportive staff. One-hundred fourteen (114; 39.4%) nurses, 50 (39.4%%) doctors, and 27 (19%) supportive staff had a post-vaccination COVID-19 infection (Table 3). Nineteen (16.5%) of HCWs from the Obstetrics and Gynaecology (OB/GYN) department had BTIs and 18 (15.7%) HCWs from the Pediatrics department (Figure 2) had BTIs.

The correlation of the profession of the participants with the prevalence of COVID-19 infections was evaluated using direct logistic regression and was statistically significant (p-value < 0.05). HCWs from 23 different departments participated and most of them were from diagnostic laboratories (17.5%).

Cycles of duty performed 

HCWs who performed one to five cycles of COVID duty were more likely to get infected when compared to those who have not done or have performed more than five cycles of COVID duty (Table 1). The correlation of COVID-19 BTI with cycles of COVID duty performed was analyzed and was statistically significant (p-value < 0.05) (Table 1). BTI was most commonly seen among the HCWs who had done one to five cycles of COVID duty (54.8%) (Table 1).

Severity of infection

Hospitalization was required for a very small number of vaccinated HCWs (19; 3%) who tested positive for COVID-19 and eight HCWs among them were due to BTI. Among HCWs working directly with patients, 116 (41.1%) of them had COVID-19 infection post-vaccination and 64 (55.7%) had BTI (Table 1), of which 4% (5/115) was severe and required hospitalization with oxygen supply; three of them had diabetes mellitus and all were symptomatic. No cases of fatality were reported in our study.

Co-morbid conditions

Eighty-eight percent (88%; 596/678) of the study population had no underlying diseases, 14 participants with diabetes mellitus had post-vaccination COVID-19 infection and 10 of them were BTI. Twenty-two participants with hypertension had post-vaccination COVID-19 infection with 13 among them being BTI (Table 1).

Vaccine dose gap

**Figure 3:**
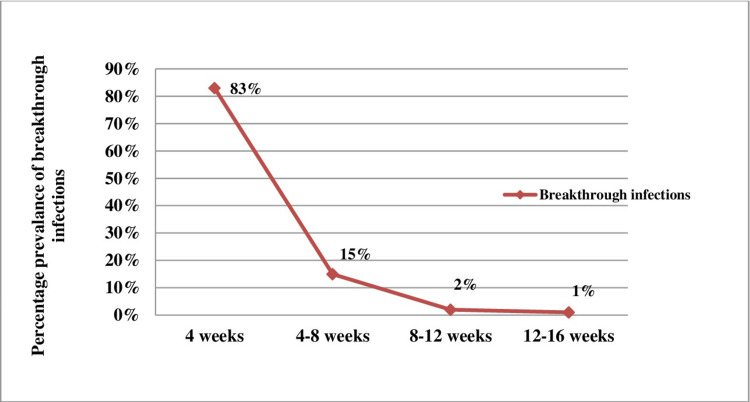
The inverse relation of breakthrough infection with a gap between two doses of the Covishield vaccine

The dose gap between the two vaccinations was four to 16 weeks. A negative correlation between breakthrough infections and dose gap was found, i.e. as the dose gap between the two vaccinations increased, the number of BTI decreased gradually (Figure 3).

## Discussion

HCWs have always battled with infectious agents causing pandemics and epidemics, and they also have the potential to spread the disease [14]. Since the beginning of this pandemic, HCWs have been at increased risk of acquiring COVID-19 infection and hospitalization [15]. Surveillance data reported by WHO between January 2020 and May 2021 showed the death of 6643 HCWs due to SARS-CoV-2 [16]. Limited data exists for SARS-CoV-2 and other infections among HCWs, as there are no standardized methods for the measurement of infections and surveillance of disease among them [17].

The prevalence of COVID-19 among HCWs in this study was 33% (226/678), 39.38%% (89/226) were positive before vaccination, and 60.6% (137/226) after vaccination. BTI was reported in 18.5% (115/ 620) of positive COVID-19 respondents. This was similar to findings reported by Doke et al. in Pune who studied HCWs after the second wave, where they reported BTIs of 16.8% [13]. Sharma et al. reported symptomatic COVID-19 infections in 16.9% of HCWs and BTI in 13.3% [18]. Mahajan et al. recorded an 11% prevalence of SARS-CoV-2 infection among HCWs in Mumbai [12]. The second wave, which began on March 13, 2021, till June 13, 2021, was driven by the delta variant (B.1.617.2) of the virus [19], which was aptly labeled by the WHO as the “fastest and the fittest” strain that had emerged till date [20]. Transmission of the delta strain was twice that of the wild-type strain, it had a reproduction number (Ro) of 5.08 and a high immune evasion, which resulted in the dominance of the Delta strain [19-21]. A nationwide study conducted by ICMR in 17 states during the second wave found that a large number (86.09%) of BTI was caused by the Delta variant (B.1.617.2) of SARS-CoV-2 in India [21]. The maximum number of confirmed BTI 37% (43/115) in our study were reported in May and 22.6% (26/115) in June 2021, the period when highly transmissible delta strain was circulating in the community. The available SARS-CoV-2 vaccine then had reduced neutralizing capability against the Delta variant and was thus one of the main causes for an increased prevalence of breakthrough infection in our study [21].

The type of vaccine used has also been implicated in causing BTI. Kameel et al., in a multicentric study in Egypt, reported a high rate of BTI of 63.5% among recipients of the inactivated COVID-19 vaccine followed by 26.5% in viral vector vaccines and 7.5% in mRNA vaccine recipients [22]. All the participants in this study had received Covishield, which is a viral vector vaccine.

Symptoms for BTI experienced by HCWs were mild and hospitalization was required for 6.95% (8/115) of HCWs with BTI and 4.3% (5/115) had severe clinical symptoms requiring supplemental oxygen. Three of the serious, hospitalized patients had diabetes mellitus as a preexisting co-morbid condition and no case fatalities were observed. In concurrence with our findings, symptoms for BTI were less severe with very few hospitalizations in the ICMR study conducted by Gupta et al., which reported only 67 cases (9.8%) of hospitalization and three (0.4%) case fatalities [21], while a multicentric study done in India by Rahi et al. reported 8.57% (6/70) of hospitalization [23], and Doke et al. in Pune recorded 23.36% (43/184) [13].

The disease-modifying nature of COVID-19 vaccines has been shown by various researchers, where only mild to less severe infections occurred in vaccinated individuals [13,21,23]. Doke et al. studied patients with BTI in two hospitals in Pune and concluded that replication of the virus is reduced in vaccinated individuals because of the immediate rise in immune response and high levels of neutralizing antibodies during BTI [13]. Thus, vaccination lowers the severity of disease, hospitalization, and mortality of BTI, which was also suggested by Gupta et al. [21]. The constant change in the policy of the Ministry of Health and Family Welfare (MOHFW), advising mild and moderate COVID-19 patients for home isolation, and restricting co-morbid HCWs from COVID-19 duties may also have contributed to decreased numbers in hospitalization.

BTI was recorded in 46% (53/670) of nurses and 23.5% (27/670) of doctors, the highest as compared to other healthcare professions. Doctors and nurses are involved in direct patient care, and aerosol-generating procedures make them vulnerable to contracting infections. Long hours of contact with symptomatic patients are a major predisposing factor for acquiring COVID-19 [24].

An increased number of HCWs with BTI were from the OB/GYN department - 17% (19/115) and the Pediatrics department - 16% (18/115). Garzaro et al., in a study on the transmission of COVID-19 infection in HCWs in Italy, have shown that physicians, despite being the main source of infection, are also the most vulnerable group [25]. They could further transmit and spread the infection as they move while attending consultations in various wards.OB/GYN department is open round the clock and pregnant women follow a set path from the emergency to the delivery room/operation theater and then the ward - all the while, with a high potential to spread infection [25]. They found a three times more risk of COVID-19 infections in HCWs in the maternity ward [25]. Marwah et al. also suggested that the nursing staff are vulnerable to infection, as they are in closer contact with patients while delivering nursing care, especially in an OB/GYN ward where constant monitoring for extended procedures is necessary [26].

Not all infected children with COVID-19 infection have symptoms, and even those with symptoms are not diagnosed in time [27], thus the likelihood of transmitting infection to HCWs when admitted for non-respiratory ailments. These cohorts of patients are unreliable maskers and do not always follow the infection control protocols, and taking care of a sick child almost always includes cuddling and touching by the parents and HCWs, in turn, increasing the risk of transmission [28].

Sensitization training in Infection control, hand hygiene, and personal protective equipment (PPE) donning and doffing were conducted regularly for each batch of staff going for COVID-19 duty but adherence and compliance were not monitored. Wotherspoon et al., in their study on compliance with PPE among HCWs in Queensland, Australia, observed that 90% made at least one mistake when donning or doffing their PPE, increasing their risk of acquiring infection [29]. Regular PPE compliance audits, hand hygiene, and bio-medical waste audits are necessary in COVID-19-dedicated areas and other critical care areas in the hospital.

Dose interval was observed to be an important influencer of COVID-19 infections. It was observed that as the dose gap between the two vaccine doses increased, the occurrence of COVID-19 infection decreased. Kaur et al., in their study on risk factors for the occurrence of COVID-19 in people who had been vaccinated, have reported that there was an increased risk of acquiring infection post-vaccination when the interval between the two doses was ≤ 30 days when compared to a dose interval of > 60 days [30]. Our report is also consistent with the exploratory findings in randomized controlled trials of the ChAdOx1 nCoV-19 (AZD1222) vaccine where the vaccine efficacy was reported to be higher after the second dose when the dose interval was longer [6].

Our study had a few drawbacks. It was a questionnaire-based study, which is at risk of response and interviewer bias, and this could not be ruled out. The study participants were recruited from only one hospital, which may have resulted in a decreased sample size.

## Conclusions

COVID-19 infection during the second wave was dominated by the Delta variant, which was highly transmissible and virulent as compared to Alpha. Vaccines provided protection against this variant and decreased morbidity and mortality among HCWs.

We have created baseline demographic data of infection for HCWs in our institute, who are at the frontline of all outbreaks and epidemics. A comprehensive health care package with regular health screening for infectious diseases for all HCWs should be made a priority by policymakers. Cases of infection in HCWs during future pandemics like in the case of SARS-CoV-2 and H1N1 and the outbreaks of the Nipah virus that occurred in Kerala and Siliguri in West Bengal will pose a serious problem for already overburdened human resource and will also have effects on transmission to the workplace and the community.
